# Effects of GHR Deficiency and Juvenile Hypoglycemia on Immune Cells of a Porcine Model for Laron Syndrome

**DOI:** 10.3390/biom13040597

**Published:** 2023-03-26

**Authors:** Marie-Christin Schilloks, Isabella-Maria Giese, Arne Hinrichs, Lucia Korbonits, Stefanie M. Hauck, Eckhard Wolf, Cornelia A. Deeg

**Affiliations:** 1 Chair of Animal Physiology, Department of Veterinary Sciences, LMU Munich, D-82152 Martinsried, Germany; 2 Chair of Molecular Animal Breeding and Biotechnology, Gene Center and Department of Veterinary Sciences, LMU Munich, D-85764 Oberschleißheim, Germany; 3 Metabolomics and Proteomics Core, Helmholtz Center Munich, German Research Center for Environmental Health, D-80939 Munich, Germany

**Keywords:** Laron syndrome, *GHR*-KO, porcine model, immune function, proteomics, PBMCs, interferon-α, hypoglycemia

## Abstract

Laron syndrome (LS) is a rare genetic disorder characterized by low levels of insulin-like growth factor 1 (IGF1) and high levels of growth hormone (GH) due to mutations in the growth hormone receptor gene (*GHR*). A *GHR*-knockout (*GHR*-KO) pig was developed as a model for LS, which displays many of the same features as humans with LS-like transient juvenile hypoglycemia. This study aimed to investigate the effects of impaired GHR signaling on immune functions and immunometabolism in *GHR*-KO pigs. GHR are located on various cell types of the immune system. Therefore, we investigated lymphocyte subsets, proliferative and respiratory capacity of peripheral blood mononuclear cells (PBMCs), proteome profiles of CD4^−^ and CD4^+^ lymphocytes and IFN-α serum levels between wild-type (WT) controls and *GHR*-KO pigs, which revealed significant differences in the relative proportion of the CD4^+^CD8α^−^ subpopulation and in IFN-α levels. We detected no significant difference in the respiratory capacity and the capacity for polyclonal stimulation in PBMCs between the two groups. But proteome analysis of CD4^+^ and CD4^−^ lymphocyte populations revealed multiple significant protein abundance differences between *GHR*-KO and WT pigs, involving pathways related to amino acid metabolism, beta-oxidation of fatty acids, insulin secretion signaling, and oxidative phosphorylation. This study highlights the potential use of *GHR*-KO pigs as a model for studying the effects of impaired GHR signaling on immune functions.

## 1. Introduction

Laron syndrome (LS) is a rare inherited disorder characterized by low circulating insulin-like growth factor 1 (IGF1) and high circulating growth hormone (GH) due to mutations in the growth hormone receptor gene (*GHR*) [1]. Features of LS include growth retardation, short stature, adolescent obesity, transient juvenile hypoglycemia, and a remarkably low incidence of cancer and diabetes [2,3]. Dysregulation of the hypothalamic–pituitary–somatotropic axis in LS patients is caused by *GHR* disruption, resulting in a lack of feedback mechanisms by IGF1 in the pituitary [4,5,6]. This causes abnormally high levels of circulating GH secreted by the pituitary gland [6].

Growth hormone receptors (GHR) are expressed on various cell types of the immune system, which makes it interesting to study the effects of disrupted GHR signaling on immune functions [7]. The effects of GH on immune cell functions are still uncertain as different and sometimes even contradictory findings were reported [8,9]. Many studies in different species have demonstrated the effects of GH on immune cell subset composition in vivo and lymphocyte proliferation in vitro [10,11,12,13,14,15,16], while some reports contradict the view that GH affects immune functions [17,18,19].

To date, the influence of GH on immune functions has been studied primarily in mouse models, but the immune systems of mice and humans differ in numerous characteristics [20,21]. One of those features is the population size of leukocyte subsets [22]. Since GHR expression is heterogeneous among leukocyte types and leukocyte subsets differ between species, disruption of *GHR* could affect immune functions in a species-specific manner [22,23]. While human leukocytes mainly consist of neutrophils, lymphocytes comprise the majority (75–90%) of leukocytes in mice [24,25]. In contrast, pigs resemble the human leukocyte composition more closely, with 27–60% lymphocytes [26,27], thus making the pig an interesting model for immunological research [20,28].

Recently, a *GHR* knockout (*GHR*-KO) pig was developed as a model for LS, resembling many features of the human disorder and providing an opportunity to study the effects of impaired GHR signaling on immune functions [29]. Moreover, *GHR*-KO pigs display transient juvenile hypoglycemia. We have already detected differences in mitochondrial respiration in immune cells of hyperglycemic pigs expressing mutant insulin C94Y (*INS*^C94Y^) which makes it interesting to study the effects of prolonged hypoglycemia in *GHR*-KO pigs on immunometabolism [30]. Fasting in humans was demonstrated to cause a shift in lymphocyte populations towards an anti-inflammatory phenotype, and the percentage of naïve CD4^+^ T cells was shown to positively correlate with insulin sensitivity in humans [31,32]. Thus, we hypothesized that prolonged hypoglycemia and enhanced insulin sensitivity in *GHR*-KO pigs may lead to enhanced immune function, especially in CD4^+^ T cells.

Therefore, this study investigated the immunometabolism of *GHR*-KO pigs and WT controls, including alterations in lymphocyte subsets, proliferative and respiratory capacity, and proteome profiles, thus extending previous studies in humans and rodents on the relationship between GH and immune functions.

## 2. Materials and Methods

### 2.1. Animal Model and Sample Preparation

In this study, samples of 30 wild-type (WT) pigs (18 females, 12 males) and 21 *GHR*-KO pigs (14 females, 7 males) at the age of 12 weeks were used. *GHR*-KO pigs were generated as previously described [29]. In order to minimize sex-specific effects, similar proportions of female and male animals in the WT and *GHR*-KO groups were used in experiments whenever this was possible. All animals were housed under controlled conditions, had free access to water, and were fed a commercial diet. For the determination of blood glucose, animals were fasted overnight. Body weights and blood glucose levels were determined immediately after blood withdrawal using a Precision Xceed glucometer with Precision XtraPlus test strips (Abbott, Wiesbaden, Germany). Fasted blood glucose levels of *GHR*-KO pigs tested in this study were consistent with earlier characterizations of this model (Supplementary Figure S1) [29]. Heparinized venous whole blood and serum samples were collected. Peripheral blood mononuclear cells (PBMCs) were isolated from whole blood by density gradient centrifugation (RT, 500× *g*, 25 min, brake off) with Pancoll separating solution (PAN-Biotech, Aidenbach, Germany), and restored in phosphate-buffered saline (PBS, pH 7.4) or RPMI medium (PAN-Biotech) supplemented with 10% heat-inactivated fetal calf serum (FCS) and 1% penicillin/streptomycin (both Biochrom, Berlin, Germany). After clotting of blood samples for 1 h at room temperature, serum was separated by centrifugation (716× *g*) for 10 min at room temperature and stored at −20 °C until further analysis. Blood withdrawal was performed according to the German Animal Welfare Act with permission from the responsible authority, Government of Upper Bavaria, following the ARRIVE guidelines and Directive 2010/63/EU. Approval numbers: ROB-55.21-54-2532-70-12; ROB-55.2-2532.Vet_02-19-195.

### 2.2. Differential Lymphocyte Count

To determine the relative proportion of lymphocyte populations, 3 μL of heparinized venous whole blood from each animal (WT: *n* = 7; 5 females, 2 males; *GHR*-KO: *n* = 6; 3 females, 3 males) was spread across the length of glass microscope slides and dried immediately. Dry slides were stained with Haema Quick-Stain Diff-Quick solutions (LT-SYS, Berlin, Germany). Under a light microscope, 100 leukocytes per slide were counted manually and identified by a veterinarian.

### 2.3. Identification of Immune Cell Subsets by Flow Cytometry

Four independent experiments with a total number of 31 animals (WT: *n* = 16, 10 females, 6 males; *GHR*-KO: *n* = 15, 10 females, 5 males) were performed. Staining of 3 × 10^5^ cells per well was performed with mouse anti-human CD79a monoclonal antibody (mab) (clone HM57, IgG1; Bio-Rad AbD Serotec, Puchheim, Germany; 1:100; cross-reactive to pig [33]) for the identification of B cells (WT: *n* = 10; *GHR*-KO: *n* = 10). Alexa Fluor 488-conjugated rat anti-human CD3ϵ mab (clone CD3-12, IgG1; 1:200; cross-reactive to pig [33]) was used for the identification of T cells (WT: *n* = 5; *GHR*-KO: *n* = 5). We used FITC-conjugated mouse anti-pig CD4α mab (clone MIL17, IgG2b; Bio-Rad AbD Serotec; 1:20) and Alexa Fluor 647-conjugated mouse anti-pig CD8α mab (clone 76-2-11, IgG2a; Becton Dickinson, Heidelberg, Germany; 1:200) to identify αβ T cells, and Alexa Fluor 647-conjugated mouse anti-pig SWC5 mab (clone b37c10, IgG1; Bio-Rad AbD Serotec; 1:5) for the identification of a γδ T cell subpopulation (WT: *n* = 6; *GHR*-KO: *n* = 5). For unconjugated primary mab, a secondary antibody was used (Alexa Fluor 647-conjugated goat F(ab’)2 anti-mouse IgG (Fc); Dianova, Hamburg, Germany; 1:500). Dead cells were excluded via labeling with Viobility 405/520 Fixable Dye (Miltenyi Biotec, Bergisch Gladbach, Germany; 1 µL/1 × 10^7^ cells) before staining with antibodies. Analyses were performed with MACSQuant Analyzer 10 (Miltenyi Biotec). Data were analyzed with Flowlogic Software (Miltenyi Biotec). The gating strategy is presented in Supplementary Figure S2.

### 2.4. Polyclonal Stimulation of PBMC

To assess the proliferative response to four different mitogens, two independent experiments with a total of 20 animals (WT: *n* = 10, 7 females, 3 males; *GHR*-KO: *n* = 10, 6 females, 4 males) were performed. Triplicates were generated for each animal and mitogen. PBMCs were either stimulated by pokeweed mitogen (PWM; Sigma-Aldrich; 1 µg/mL), concanavalin A (ConA; Sigma-Aldrich; 1 µg/mL), phytohemagglutinin-L (PHA-L; PAN-Biotech; 1 µg/mL) or *M. paradisiaca* lectin (BanLec; L1410; Biozol, Eching, Germany, 1 µg/mL). Control PBMCs remained unstimulated. After incubation for 32 h at 37 °C, cells were incorporated with ^3^H-thymidine (Perkin Elmer, Hamburg, Germany) and incubated for an additional 16 h. After harvesting, the incorporation of ^3^H-thymidine was quantified by measuring counts per minute (cpm) using a microbeta counter (Perkin Elmer). The proliferation rate was expressed as the ratio of ^3^H-thymidine incorporation by stimulated cells compared with unstimulated cells.

### 2.5. Magnetic Activated Cell Sorting of CD4^−^ and CD4^+^ PBMC

A total of 6 × 10^7^ PBMCs from 5 WT pigs (3 females, 2 males) and 4 *GHR*-KO pigs (2 females, 2 males) were incubated in staining buffer with mouse anti-pig CD4α mab (clone MIL17, IgG2b; Bio-Rad AbD Serotec; 1:50) at 4 °C for 20 min. Staining buffer contained PBS (pH 7.2) and was supplemented with 2 mM EDTA and 0.5% bovine serum albumin (BSA). Cells were then resuspended in staining buffer before adding anti-mouse IgG2a/b MicroBeads (Miltenyi Biotec; 20 μL per 10^7^ total cells) for an incubation time of 15 min. Next, magnetic separation was performed using LS columns (Miltenyi Biotec). Magnetically labeled CD4^+^ PBMCs were retained in the magnetic field of the columns, while unlabeled flow-through (CD4^−^ PBMCs) passed through the columns in three washing steps. The CD4^+^ PBMCs fraction was eluted by removing the column from the magnetic field and flushing it with staining buffer. For filter-aided sample preparation (FASP), 6 × 10^5^ positively selected cells of eluate and flow-through were each pelleted and stored at −20 °C. The isolation of porcine CD4^+^ PBMCs, which consist of naïve CD4^+^ T helper cells, activated/memory T helper cells, and plasmacytoid dendritic cells, routinely achieved 95% ± 1% SD purity as confirmed by flow cytometry. Flow-through (CD4^−^ PBMCs) consisted of monocytes, natural killer (NK) cells, B cells, γδ-T cells, naïve and memory CD8^+^ cytolytic T cells, and natural killer T (NKT) cells.

### 2.6. Sample Digestion

A total of 6 × 10^5^ cells per sample were digested using a modified FASP procedure [34,35]. After reduction and alkylation using dithiothreitol and iodoacetamide, the proteins were centrifuged on a 30 kDa cutoff filter device (Sartorius, Göttingen, Germany), washed twice with urea buffer (UA buffer; 8 M urea in 0.1 M Tris/HCl, pH 8.5) and twice with 50 mM ammonium bicarbonate. The proteins were digested for two hours at room temperature using 0.5 µg Lys-C (Wako Chemicals, Neuss, Germany) and for 16 h at 37 °C using 1 µg trypsin (Promega, Mannheim, Germany). After centrifugation (10 min at 14,000× *g*) the eluted peptides were acidified with 0.5% trifluoroacetic acid and stored at −20 °C.

### 2.7. Differential Proteome Analysis by LC MS/MS

Alterations in protein abundances in CD4^+^ PBMCs and CD4^−^ PBMCs of 5 WT pigs (3 females, 2 males) and 4 *GHR*-KO pigs (2 females, 2 males) were analyzed. Acidified eluted peptides were analyzed in the data independent acquisition (DIA) mode on a Q Exactive HF-X mass spectrometer (Thermo Fisher Scientific, Waltham, MA, USA) online coupled to an ultra-high-performance liquid chromatography (UHPLC) system (Ultimate 3000, Thermo Fisher Scientific). Tryptic peptides were automatically loaded on a C18 trap column (300 µm inner diameter (ID) × 5 mm, Acclaim PepMap100 C18, 5 µm, 100 Å, LC Packings) at 30 µL/min flow rate prior to C18 reversed-phase chromatography on the analytical column (nanoEase MZ HSS T3 Column, 100 Å, 1.8 µm, 75 µm × 250 mm, Waters, Eschborn, Germany) at 250 nL/min flow rate in a 95 min non-linear acetonitrile gradient from 3 to 40% in 0.1% formic acid. The data-independent acquisition method consisted of a survey scan from 300 to 1650 mass-to-charge ratio at 120,000 resolution and an automatic gain control (AGC) target of 3e^6^ or 120 ms maximum injection time. Fragmentation was performed via higher-energy collisional dissociation with a target value of 3e^6^ ions determined with predictive AGC. Precursor peptides were isolated with 37 variable windows spanning from 300 to 1650 mass-to-charge ratio at 30,000 resolution with an AGC target of 3e^6^ and automatic injection time. The normalized collision energy was 28, and the spectra were recorded in profile mode.

For label-free quantification of DIA data, the DIA LC-MS/MS data set was analyzed by comparing the MS2 fragment spectra from the recorded windows against a spectral library collected from data-dependent acquisition data (54 raw files, derived from high pH fractionated porcine granulocytes and lymphocytes) from the same instrument. The spectral library was generated directly in Spectronaut Pulsar XII (Biognosys, Schlieren, Switzerland) as described [36]. Spectronaut was equipped with the Ensembl Pig database (Release 75 (Sscrofa10.2), 25,859 sequences, https://www.ensembl.org, accessed on 8 December 2022). The default settings for the database match included: full trypsin cleavage, peptide length between seven and fifty-two amino acids, and maximally two missed cleavage sites. Carbamidomethylation of cysteine was set as a fixed modification, and the only variable modifications allowed were deamidation and oxidation of methionine. All false discovery rates (FDRs) were set as 0.01 for the peptide-spectrum match (PSM), peptide and protein. The best 3–6 fragments per peptide were included in the library. The final spectral library generated in Spectronaut contained 5537 protein groups and 84,044 peptide precursors. Quantification was based on cumulative MS2 area levels. Briefly, raw files were imported into Spectronaut, and XIC extraction settings were set to dynamic with a correction factor of one. Normalization was performed by default settings in Spectronaut, based on the local regression normalization [37]. Automatic calibration mode was chosen, and interference correction on MS1 and MS2 levels was enabled. Peptide and protein identification was filtered to satisfy an FDR of 1% by the mProphet approach [38]. Only proteotypic peptides were considered for protein quantification, applying summed precursor quantities based on MS2 area quantity. A match between runs was enabled with the q-value percentile mode 0.2 threshold. Thus, only peptide precursors that passed the q-value cut-off in at least 20% of samples were reported. For the following evaluation of data, statistical analysis was performed on log2 transformed normalized abundance values using Student’s *t*-test. All significant, differentially abundant proteins quantified with at least two peptides were subsequently included in the bioinformatic analysis. As DIA proteomics provides high quantification precision [39], biological cutoffs were not applied in bioinformatic analysis. Differences in protein abundance with *p* ≤ 0.05 were considered significant.

### 2.8. Bioinformatic Analysis

In pathway enrichment analysis with open-source software ShinyGO [40] (http://bioinformatics.sdstate.edu/go/ version 0.77, accessed on 8 February 2023), the FDR cutoff was set to 1%. Analysis was performed on the best-matched species (human). The *p*-value for enrichment analysis was calculated using hypergeometric distribution followed by FDR correction. In pathway enrichment analysis with Ingenuity Pathway Analysis (IPA; Qiagen, Hilden, Germany, https://digitalinsights.qiagen.com/ accessed on 8 December 2022), bioinformatic analysis was performed on human orthologues. Z-score describes the prediction of activation or inhibition of the respective pathway. The significance threshold was set to –log (*p*-value) > 1.3. IPA analyzes the overrepresentation of proteins from data input in canonical pathways deposited in the IPA library, as previously described [41]. This allows insight into the possible physiological effects of upstream molecules on these proteins and their allocation to downstream pathways.

### 2.9. Real-Time Cell Metabolic Analysis by Seahorse XFe Analyzer

Metabolic phenotypes of PBMCs from 7 WT pigs (5 females, 2 males) and 6 *GHR*-KO pigs (4 females, 2 males) were determined in three independent experiments using a Seahorse XFe Analyzer (Agilent Technologies, Waldbronn, Germany). Oxygen consumption rate (OCR) was measured, indicating mitochondrial respiration and extracellular acidification rate (ECAR) reflected glycolysis [42]. Sterile XF assay buffer (Seahorse XF RPMI medium supplemented with 10 mM glucose, 2 mM L-glutamine, and 1 mM pyruvate, pH 7.4; Agilent Technologies) was used for the experiments according to the manufacturer’s instructions. Before starting the assay, sensor cartridges (Agilent Technologies) were prepared to which oligomycin, carbonyl cyanide-4-(trifluoromethoxy)-phenylhydrazone (FCCP), and rotenone were added along with antimycin A. The cartridges were then used for the experiments. A total of 1 × 10^6^ PBMCs were seeded in 24-well XF24 cell culture microplates (Agilent Technologies), while four wells were kept free from cells for background correction. Baseline OCR and ECAR were measured before adding oligomycin, FCCP, and rotenone together with antimycin A. OCR was reported in units of pmol/minute and ECAR in mpH/minute.

### 2.10. Determination of IFN-*α Concentrations*

Porcine interferon-alpha (IFN-α) concentration was determined in the serum of 16 WT pigs (10 females, 6 males) and 16 *GHR*-KO pigs (10 females, 6 males). Serum was stored at −20 °C until usage. A porcine IFN-α ELISA kit (RAB1131; Sigma-Aldrich, Taufkirchen, Germany) was used and the absorbance was detected at an optical density of 405 nm using a Tecan Sunrise microplate reader (Tecan, Crailsheim, Germany).

### 2.11. Statistical Analysis

For statistical analysis of body weights, blood glucose levels, lymphocyte counts, flow cytometry data, enzyme-linked immunosorbent assay (IFN-α), polyclonal stimulation with mitogens and real-time cell metabolic assay, the Kolmogorov–Smirnov (KS) test was used first to determine Gaussian distribution. If the KS test indicated *p* < 0.05 (no normal distribution), the Mann–Whitney U test was used for statistical analysis, whereas Student’s *t*-test was used when the KS test indicated *p* > 0.05 (normal distribution). Multiple hypothesis testing correction was performed using the Benjamini–Hochberg procedure for the calculation of adjusted *p*-values. Probabilities were considered significant at *p* ≤ 0.05. (* = *p* ≤ 0.05, ** = *p* ≤ 0.001, ns = not significant).

## 3. Results

### 3.1. Knockout of Growth Hormone Receptor Did Not Affect Lymphocyte Population Percentage

**Figure 1:**
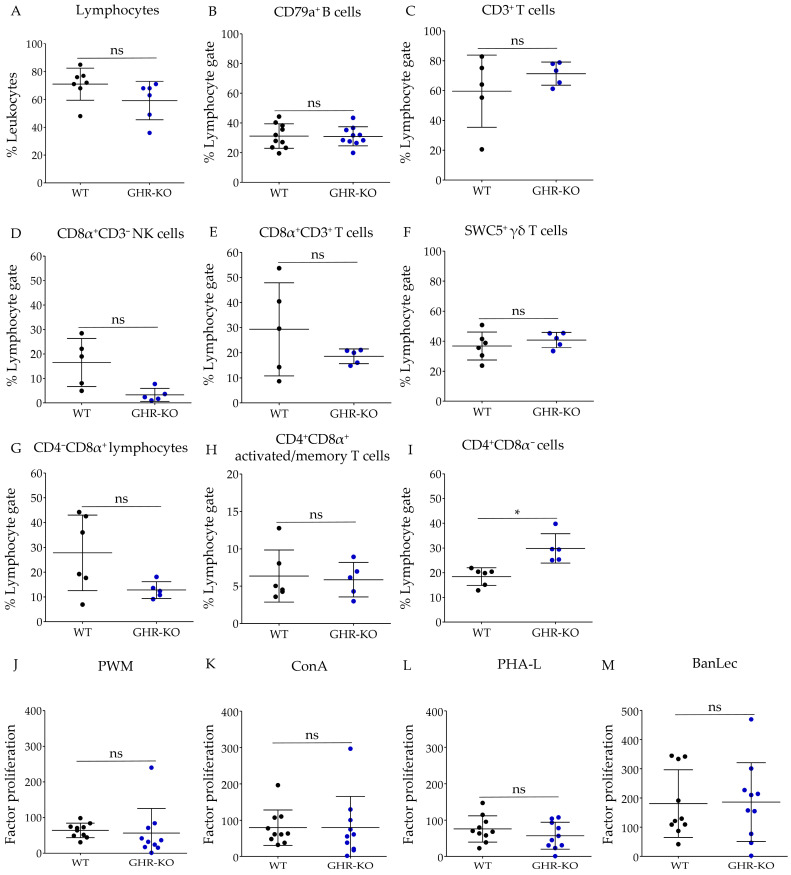
Lymphocyte populations, lymphocyte subpopulations and proliferative capacities of PBMC of WT and *GHR*-KO pigs. (**A**) Percentage of lymphocytes in the leukocytes of wild-type (WT) (black dots, *n* = 7) and growth hormone receptor knockout (*GHR*-KO) pigs (blue dots, *n* = 6). Lymphocyte percentages were not significantly different between WT and *GHR*-KO pigs. (**B**–**I**) Lymphocyte subpopulations of WT (black dots, *n* = 16) and *GHR*-KO pigs (blue dots, *n* = 15). Proportions of (**B**) CD79a^+^ B cells, (**C**) CD3^+^ T cells (**D**) CD8α^+^CD3^−^ NK cells, (**E**) CD8α^+^CD3^+^ T cells, (**F**) SWC5^+^ γδ T cells, (**G**) CD4^−^CD8α^+^ lymphocytes, (**H**) CD4^+^CD8α^+^ activated/memory T cells did not significantly differ between WT and *GHR*-KO pigs. CD4^+^CD8α^−^ cells (**I**) significantly differed between WT and *GHR*-KO pigs. (**J**–**M**) Proliferative capacity of peripheral blood mononuclear cells (PBMCs) of WT (black dots, *n* = 10) and *GHR*-KO pigs (blue dots, *n* = 10) after polyclonal stimulation with (**J**) pokeweed mitogen (PWM), (**K**) concanavalin A (ConA), (**L**) phytohaemagglutinin-L (PHA-L) and (**M**) *M. paradisiaca* lectin (BanLec) in vitro revealed similar proliferative capacity of WT and *GHR*-KO PBMCs. Data are shown as mean ± SD; ns = not significant, * *p* ≤ 0.05.

To investigate the composition of leukocytes in *GHR*-KO pigs, we first examined the relative number of peripheral blood lymphocytes (PBL) in WT and *GHR*-KO pigs. The WT pigs displayed 71 ± 4.4% lymphocytes, and the *GHR*-KO pigs 59.2 ± 5.6% lymphocytes. There was no significant difference in the relative proportion of lymphocytes (Figure 1A).

### 3.2. CD4^+^CD8α^−^ Subpopulation Ratio Differed Significantly between WT and GHR-KO Pigs

The lack of GHR signaling and the resulting low IGF1 levels may affect only certain subsets of lymphocytes. Therefore, we examined the PBL of *GHR*-KO pigs in more detail. Lymphocyte subpopulation percentages from WT and *GHR*-KO pigs were analyzed by flow cytometry (Figure 1). Populations of CD79a^+^ B cells (Figure 1B) and CD3^+^ T cells (Figure 1C) were similar between WT and *GHR*-KO pigs. Percentages of CD8^+^CD3^−^ NK cells (Figure 1D), CD8^+^CD3^+^ T cells (Figure 1E), SWC5^+^ γδ T cells (Figure 1F), CD4-CD8α^+^ lymphocytes (Figure 1G), as well as CD4^+^CD8α^+^ activated/memory T cells (Figure 1H), revealed no statistically significant difference in lymphocyte subpopulations in WT and *GHR*-KO pigs. CD4^+^CD8α^−^ cells significantly differed between WT and *GHR*-KO pigs (*p* = 0.02). While WT pigs displayed 18.4 ± 3.6% CD4^+^CD8α^−^ cells, *GHR*-KO pigs displayed 29.8 ± 5.9% CD4^+^CD8α^−^ cells (Figure 1I).

### 3.3. PBMC of WT and GHR-KO Pigs Show Similar Capacity for Polyclonal Stimulation

Because we observed a greater proportion of CD4^+^CD8α^−^ cells in flow cytometry in *GHR*-KO pigs, we wanted to determine whether these lead to aberrant proliferative capacity in *GHR*-KO pigs. Therefore, we investigated the differences in the proliferation response of PBMCs from WT and *GHR*-KO pigs after polyclonal in vitro stimulation with the four different mitogens: PWM, ConA, PHA-L, and BanLec. To evaluate the toll-like receptor-based proliferation response, we used the B- and T-cell mitogen Pokeweed mitogen (PWM) [43,44] (Figure 1J). To evaluate the proliferation ability of T cells, we used T cell mitogens concanavalin A (ConA) [45] (Figure 1K), phytohaemagglutinin-L (PHA-L) [46] (Figure 1L), and *M. paradisiaca* lectin (BanLec) [47] (Figure 1M). ConA and PHA induce proliferation of human T cells by binding to cell surface carbohydrates on glycoproteins like the TCR/CD3 complex [48]. BanLec induces proliferation via the IL-2 pathway and ELF1 in pig PBL [47]. The proliferation response in both groups was highest in response to BanLec (Figure 1M) compared to the other three mitogens. PBMCs from WT and *GHR*-KO pigs showed the same capacity after polyclonal stimulation to all tested mitogens at 12 weeks of age.

### 3.4. CD4^+^ PBMC and CD4^−^ PBMC of WT and GHR-KO Pigs Show Divergent Proteomic Profiles

**Figure 2:**
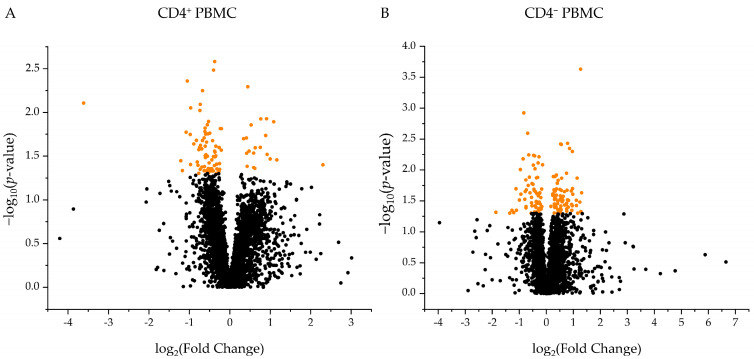
Volcano plot illustrating 3549 quantified proteins (≥2 unique peptides) in (**A**) CD4^+^ PBMCs and (**B**) CD4^−^ PBMCs of *GHR*-KO/WT pigs. Log2 abundance differences between groups (*x*-axis) are plotted against log10 statistical significance values (*y*-axis). Proteins that were significantly (*p* ≤ 0.05) differentially abundant between WT and *GHR*-KO pigs are shown in orange, while proteins below the significance threshold are displayed in black.

To gain deeper insight into the immune function of *GHR*-KO pigs at the molecular level, we characterized the proteomes of CD4^+^ PBMCs and CD4^−^ PBMCs from WT and *GHR*-KO pigs, using differential proteomic analysis. The percentage of CD4^+^ cells in the PBMCs of WT pigs (*n* = 6; 3 females, 3 males) was 23.3 ± 2.1%. In *GHR*-KO pigs (*n* = 5; 4 females, 1 male) CD4^+^ cell percentage in PBMCs was 28.3 ± 1.7%. CD4^+^ cell percentages in PBMCs did not significantly differ between WT and *GHR*-KO pigs. A high-resolution proteome was obtained with a total of 4295 identified proteins, of which 3549 were quantified with at least two unique peptides. In CD4^+^ PBMCs, 93 proteins were significantly (*p* ≤ 0.05) differentially expressed between *GHR*-KO and WT pigs (Figure 2A). In CD4^−^ PBMCs, 139 proteins were significantly (*p* ≤ 0.05) differentially expressed (Figure 2B).

### 3.5. CD4^+^ PBMC of WT and GHR-KO Pigs Show Divergent Proteomic Profiles Pointing to Deviant Amino Acid Metabolism in GHR-KO Pigs

**Figure 3:**
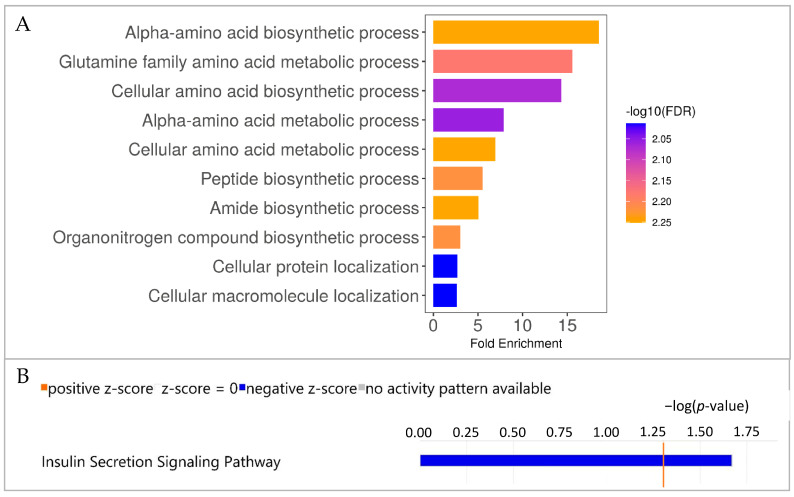
Pathway enrichment analyses of divergent proteomic profiles in CD4^+^ PBMC of *GHR*-KO/WT pigs. (**A**) Pathway enrichment analysis using ShinyGO software showing the 10 pathways with the highest fold enrichment. Enrichment analysis of differentially abundant proteins in CD4^+^ PBMCs revealed the highest enrichment of “Alpha-amino acid biosynthetic process” and in *GHR*-KO pigs. The length of the bars represents the fold enrichment, and the color of the bars represents the FDR. (**B**) Pathway enrichment analysis using Ingenuity Pathway Analysis (IPA). The bar chart shows all enriched pathways with a prediction for activation or inactivation. Negative (blue) z-score indicates inactivation predicted by IPA. Color intensity correlates with z-score, while bar length indicates statistical significance. The orange, vertical line represents the –log (*p*-value) threshold which was 1.3.

To interpret the observed differences in protein abundances between WT and *GHR*-KO pigs, pathway enrichment analysis was performed. Analysis of proteins in CD4^+^ PBMCs that differed significantly (*p* ≤ 0.05) in abundance between WT and *GHR*-KO pigs using ShinyGo software pointed to differences in the metabolism of these cells (Figure 3A). Enrichment analysis of the 93 differentially expressed proteins in CD4^+^ PBMCs showed the highest enrichment of “Alpha-amino acid biosynthetic process” in *GHR*-KO pigs pointing to divergent metabolism of *GHR*-KO vs. WT CD4^+^ PBMCs. Proteins included in this pathway are listed in Table 1. IPA analysis of our data set revealed a total of 34 significantly enriched canonical pathways in CD4^+^ PBMCs, although most of these pathways did not predict activity patterns. No pathway was predicted to be activated in *GHR*-KO CD4^+^ PBMCs, but the “Insulin Secretion Signaling Pathway” was predicted to be less active in *GHR*-KO vs. WT cells (Figure 3B). Proteins included in the “Insulin Secretion Signaling Pathway” are listed in Table 1.

### 3.6. CD4^−^ PBMC from WT and GHR-KO Pigs Show Divergent Proteomic Profiles, Indicating Deviant Fatty Acid Metabolism in GHR-KO Pigs

**Figure 4:**
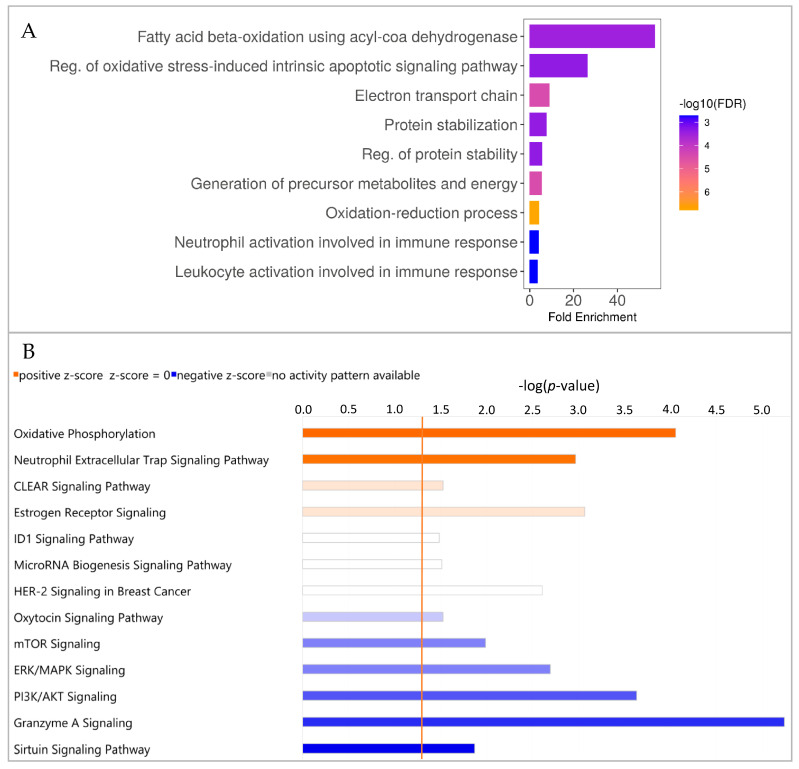
Pathway enrichment analyses of divergent proteomic profiles in CD4^−^ PBMC of *GHR*-KO/WT pigs. (**A**) Pathway enrichment analysis using ShinyGO software showing the 10 pathways with the highest fold enrichment. Enrichment analysis of the differentially abundant proteins in CD4^−^ PBMCs revealed the highest enrichment of “Fatty acid beta-oxidation using acyl-CoA dehydrogenase”. Bar length represents fold enrichment, and bar color represents FDR. (**B**) Pathway enrichment analysis using IPA. The bar chart shows all enriched pathways with a prediction for activation or inactivation. Negative (blue) z-score indicates inactivation predicted by IPA. Positive (orange) z-score indicates activation predicted by IPA. Color intensity correlates with z-score, while bar length indicates statistical significance. The orange, vertical line represents the –log (*p*-value) threshold, which was 1.3.

Of the 3549 proteins quantified with at least two unique peptides, 139 proteins were significantly (*p* ≤ 0.05) differentially abundant in CD4^−^ PBMCs. To characterize the CD4^−^ PBMCs from *GHR*-KO pigs, pathway enrichment analysis of the 139 proteins that were significantly (*p* ≤ 0.05) differentially abundant in CD4^−^ PBMCs was performed using ShinyGo software (Figure 4A). The signaling pathways with the highest fold enrichment in CD4^−^ PBMCs from *GHR*-KO pigs were related to metabolism. The pathway “Fatty acid beta-oxidation using acyl-CoA dehydrogenase” was highly enriched, indicating aberrant metabolism of CD4^−^ PBMCs from *GHR*-KO pigs (Figure 4A). Proteins included in this pathway are listed in Table 2. In the IPA analysis, 42 canonical pathways did not allow prediction of the activity pattern, but 13 pathways with predictions of activation or inactivation were detected (Figure 4B). IPA analysis revealed that the canonical pathway “Oxidative phosphorylation” was strongly activated and the canonical pathway “Sirtuin signaling” was inactivated in CD4^−^ PBMCs from *GHR*-KO pigs. Proteins included in the “Oxidative phosphorylation” pathway are listed in Table 2.

### 3.7. Similar Basal, ATP-Linked and Maximal Respiration of PBMC of WT and GHR-KO Pigs

**Figure 5:**
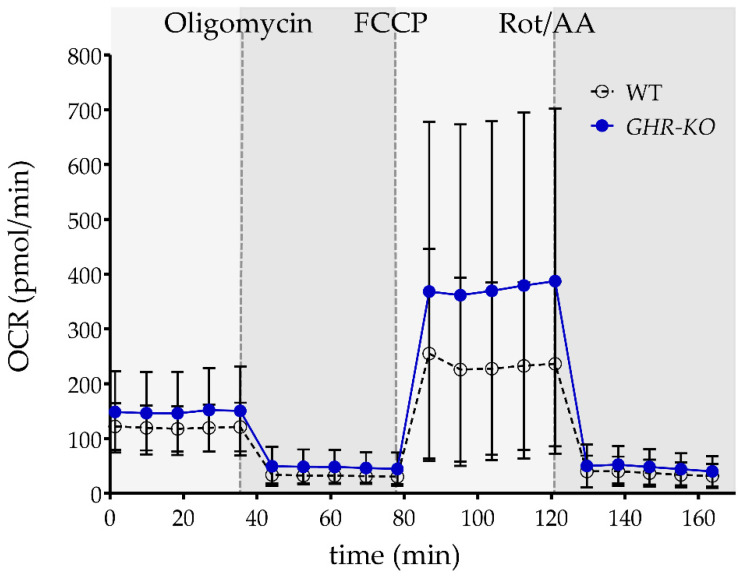
Mitochondrial respiratory profiles of PBMCs from pigs. The oxygen consumption rate (OCR) of PBMCs from WT (*n* = 7, white dots) and *GHR*-KO pigs (*n* = 6, blue dots) was measured under basal conditions and after injection of oligomycin, carbonyl cyanide-4 (trifluoromethoxy) phenylhydrazone (FCCP), rotenone, and antimycin A (Rot/AA). Compared with PBMCs from WT pigs, PBMCs from *GHR*-KO pigs showed similar basal respiration, ATP-linked respiration, maximal respiration, and spare respiratory capacity. Data are presented as means ± SD.

Because pathway analysis predicted activation of oxidative phosphorylation in CD4^−^ PBMCs of *GHR*-KO pigs, we were interested to see if the metabolic phenotype of immune cells differed between WT and *GHR*-KO pigs. All proteins involved in the canonical pathway “Oxidative phosphorylation” were associated with respiratory chain complexes (Table 2), so we decided to investigate the mitochondrial function of PBMCs via the Seahorse XF Cell Mito Stress Assay. Basal respiration, non-mitochondrial oxygen consumption, ATP-linked respiration, maximal respiration, and spare respiratory capacity, which is a measure of the ability of the cell to respond to increased energy demand or under stress, were similar between PBMCs of WT and *GHR*-KO pigs [42] (Figure 5).

### 3.8. Similar Glycolysis of PBMC of WT and GHR-KO Pigs

**Figure 6:**
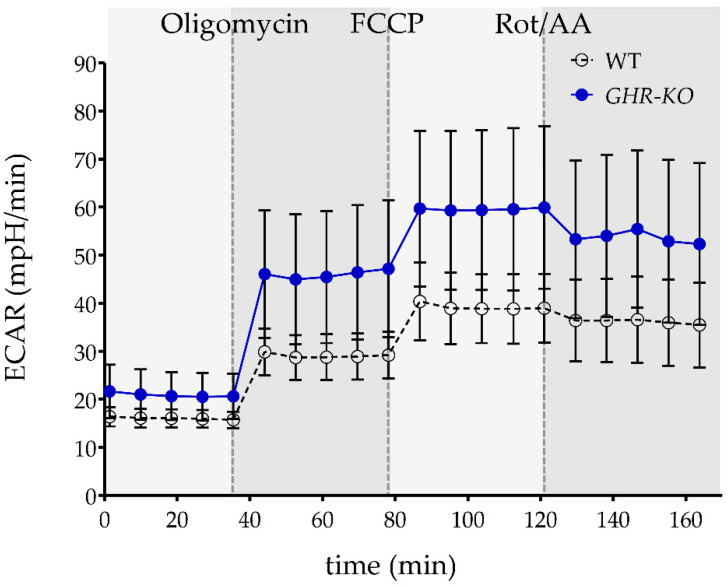
The extracellular acidification rate (ECAR) of PBMCs from WT pigs (*n* = 7, white dots) and *GHR*-KO pigs (*n* = 6, blue dots), indicating glycolysis after injection of oligomycin, FCCP, and Rot/AA, yielded similar ECAR, indicating similar glycolysis activity in WT and *GHR*-KO PBMC. Data are presented as mean ± SD.

Extracellular acidification rate, indicating glycolysis of cells, was measured at baseline and after inhibition of mitochondrial complexes (Figure 6). There was no significant difference in extracellular acidification rate (ECAR) between PBMCs of WT and *GHR*-KO pigs, indicating no difference in glycolytic reserve/capacity.

### 3.9. Differentially Abundant Protein in CD4^+^ PBMC of WT and GHR-KO Pigs Is Associated with Protein Glycosylation

Among the eight proteins whose abundance was significantly (*p* ≤ 0.01) lower in CD4^+^ PBMCs of *GHR*-KO pigs compared with WT pigs, the protein with the lowest abundance ratio was polypeptide N-acetylgalactosaminyltransferase (GALNT1), with an enrichment ratio of 0.1 (Table 3). In humans, GALNT1 is involved in the O-glycosylation of serine and threonine residues of proteins [49,50].

### 3.10. Differential Abundance of Protein FAH in CD4^+^ PBMC of WT and GHR-KO Pigs Is Related to Amino Acid Metabolism

Only one protein, fumarylacetoacetate hydrolase (FAH), showed significantly (*p* ≤ 0.01) higher abundance in CD4^+^ PBMCs of *GHR*-KO pigs compared to WT pigs with a ratio of 1.4. In humans, FAH catalyzes the final step of tyrosine degradation [51] (Table 4).

### 3.11. Differentially Abundant Protein in CD4^−^ PBMC of WT and GHR-KO Pigs Is Related with Interferon Signaling

In CD4^−^ PBMCs, 12 proteins had significantly (*p* ≤ 0.01) lower abundances in *GHR*-KO pigs compared to WT pigs. The protein with the lowest abundance ratio in CD4^−^ PBMCs from *GHR*-KO pigs (Table 5) was interferon-induced protein 44 (IFI44) with an abundance ratio of 0.5 compared to WT pigs. This was of particular interest because of its role in promoting or decreasing IFN-α signaling, thereby modulating the virus-host response by increasing or decreasing human cell proliferation [52,53,54].

### 3.12. Differentially Abundant Proteins in CD4^−^ PBMC of WT and GHR-KO Pigs Were Associated with Cell Adhesion

In CD4^−^ PBMCs of *GHR*-KO pigs (Table 6), six proteins displayed significantly (*p* ≤ 0.01) higher abundance in CD4^−^ PBMCs of *GHR*-KO pigs compared to WT pigs. Of those six proteins, integrin subunit alpha M (ITGAM) had the highest different abundance ratio in *GHR*-KO pigs (2.4) compared to WT pigs. ITGAM is a leukocyte antigen marker in pigs that is expressed, among others, on NK cells and B cells [55,56].

### 3.13. IFN-*α* Concentration in Serum of GHR-KO Pigs Was Significantly Higher

**Figure 7:**
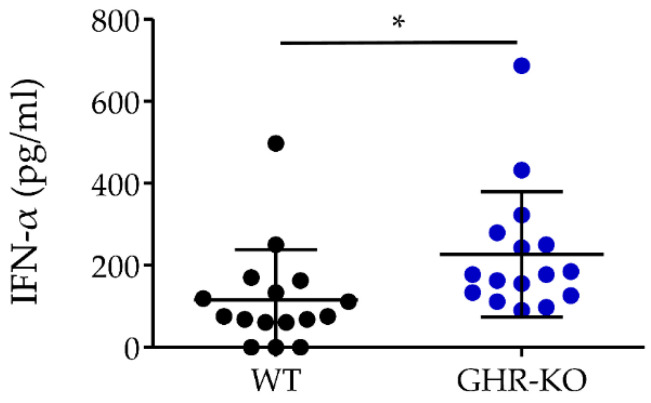
Interferon-α (IFN-α) concentration in pg/mL in sera from WT (black dots, *n* = 16) and GHR-KO pigs (blue dots, *n* = 16). *GHR*-KO pigs had significantly higher serum concentrations of IFN-α. Data are expressed as mean ± SD (* = *p* ≤ 0.05).

Because the significantly higher protein abundance of IFI44 in WT pigs compared with *GHR*-KO pigs indicated a possible difference in IFN-α secretion, we quantified IFN-α concentration in sera from *n* = 16 WT and *n* = 16 *GHR*-KO pigs. In the WT group, three samples had values below the detection limit of the assay (36 pg/mL) and were therefore set to zero in the statistical analysis. Compared to the WT pigs, the *GHR*-KO pigs showed significantly (*p* = 0.02) higher serum concentrations of IFN-α (Figure 7).

## 4. Discussion

Porcine lymphocytes express mRNA for the growth hormone receptor [57]. Furthermore, since these cells are highly dependent on glucose, the purpose of this study was to investigate how the absence of GHR signaling and prolonged hypoglycemia affect the development of the immune system in *GHR*-KO pigs [23,58,59].

When we examined the white blood cells of *GHR*-KO pigs, there was no difference in the percentage of lymphocytes between *GHR*-KO and WT pigs, and the lymphocyte percentages were consistent with previous observations in two-to-three-month-old pigs [60]. A similar lymphocyte percentage in *GHR*-KO and WT pigs suggests that reduced GHR signaling and hypoglycemia did not affect the development of lymphocytes in the bone marrow and thymus [61]. In addition, the proliferative capacity of lymphocytes from *GHR*-KO and WT pigs in response to mitogens was similar. In contrast, two patients with LS, a 19-year-old boy and a 9-year-old girl, were previously found to have decreased proliferative capacity of lymphocytes in response to PHA [16]. A possible explanation for these discrepant observations may be the fact that the *GHR*-KO and WT pigs in our study were only 12 weeks old and therefore not yet at puberty, which occurs in pigs around postnatal day 165 [62]. During puberty, GH levels double in humans [63]. In the study revealing a decreased lymphocyte proliferative capacity in a 19-year-old LS patient, children in the healthy control group were up to 15 years old, presumably in puberty, when the difference in GHR signaling between healthy humans and LS patients is greatest [16]. Thus, possible differences in lymphocyte proliferation between *GHR*-KO and WT pigs might become evident during or after puberty. Since we also wanted to study the effects of hypoglycemia, which occurs transiently in juvenile but disappears in adult *GHR*-KO pigs [29], we decided to study lymphocyte proliferation in 3-month-old animals. Nevertheless, it would be interesting to study lymphocyte proliferation in older *GHR*-KO pigs as well, independent of hypoglycemia.

Another possible reason for different results regarding the proliferative capacity of lymphocytes of LS patients and *GHR*-KO pigs could be the composition of the PBMC subsets studied. The percentage of lymphocyte subsets was altered in *GHR*-KO pigs. When Pescovitz et al. compared the proliferative capacity of CD4^+^CD8α^+^ and CD4^+^CD8α^−^ lymphocytes from pigs in response to PHA, they found a higher proliferative capacity of the CD4^+^CD8α^−^ lymphocytes [64]. Therefore, the higher percentage of CD4^+^CD8α^−^ cells in PBMCs of *GHR*-KO pigs might explain why—in contrast to LS patients—the proliferative response to mitogens was not decreased.

The pigs investigated in our study were housed together under standardized, specified pathogen-free conditions and most of them were littermates to minimize the influence of differences in antigen contact and genetic predisposition. In contrast, the LS patients analyzed by Caruso-Nicoletti et al. [16] were not directly related to the healthy control subjects.

When the lymphocyte subsets of *GHR*-KO and WT pigs were examined, the percentages of NK cells, B cells, and T cells (CD3^+^) did not differ significantly between the groups, in line with their similar age and antigen exposure, factors known to be associated with differences in lymphocyte subsets in pigs [65,66,67]. The proportions of CD3^+^ T cell subsets (SWC5^+^ γδ T cells and CD4^+^CD8α^+^ activated/memory T helper cells) were also similar in *GHR*-KO and WT pigs. CD4^+^ T cells are the primary T cell subset involved in the porcine immune response to bacteria [68,69,70,71,72,73]. Similar percentages of CD4^+^CD8α^+^ activated/memory T helper cells in *GHR*-KO and WT pigs argue against an impaired immune defense against bacteria in GHR deficiency.

In contrast, the percentage of CD4^+^CD8α^−^ cells was significantly higher in *GHR*-KO pigs. Studies in two LS patients revealed higher absolute blood CD4^+^ T cell counts compared to healthy individuals, suggesting an effect of GH on circulating CD4^+^ T cell counts [16]. In addition, higher proportions of naïve CD4^+^ T cells and reduced populations of effector/memory T cells were found in the spleens of a mouse model for LS at age 36 months [74]. Higher relative proportions of blood CD4^+^ T cells were detected in 18-month-old mice lacking growth hormone-releasing hormone, and higher percentages and absolute numbers of blood CD4^+^ lymphocytes were detected in GH-deficient humans [75,76]. Taken together, this indicates that reduced GHR signaling can lead to higher relative and absolute numbers of CD4^+^ T lymphocytes. The CD4^+^CD8α^−^ lymphocyte cluster in our pig study consists of naïve T cells and plasmacytoid dendritic cells [77,78]. It will thus be interesting to study which of the two subsets is altered.

Next, we investigated differences in the proteomes of CD4^+^ and CD4^−^ PBMCs of *GHR*-KO and WT pigs as a hypothesis-generating approach. For this purpose, CD4^+^ cells (=CD4^+^ PBMCs: naïve and activated/memory T helper cells, plasmacytoid dendritic cells) were separated from blood and, as a further population, the remaining non-CD4^+^ cells (=CD4^−^ PBMCs: monocytes, NK cells, B cells, γδ-T cells, naïve and memory CD8^+^ cytolytic T cells, and NKT cells) were analyzed. The comprehensive proteomic data set includes 4295 proteins of which 3549 were identified with ≥2 peptides. Of these, 27 proteins displayed highly significant (*p* ≤ 0.01) abundance differences between the groups.

Among the proteins with significantly reduced abundance in CD4^−^ PBMCs of *GHR*-KO pigs was interferon-induced protein 44 (IFI44). Its role in humans with LS and animal models of LS is unknown to our knowledge; however, in mice, the expression of IFI44 was demonstrated to be reduced in response to fasting in white adipose tissue, liver, and skeletal muscle [79]. Thus, hypoglycemia in *GHR*-KO pigs might contribute to the reduced IFI44 protein abundance in CD4^−^ PBMCs. The role of IFI44 in porcine immune cells is largely unknown, and its expression in porcine CD4^−^ PBMCs has, to our knowledge, not been reported; however, increased expression of IFI44 was previously detected in the spleens of pigs after virus infections, suggesting a role in immune defense against viruses [80].

As IFI44 is an interferon-induced gene [52], we determined the IFN-α levels of GHR-KO and WT pigs. In spite of reduced IFI44 expression, circulating IFN-α levels were increased in *GHR*-KO pigs. A potential reason is their hypoglycemia, since fasting was demonstrated to increase IFN-α levels in mice [81] and low fasting plasma glucose was shown to correlate with high IFN-α levels in humans [82]. Future studies should investigate IFN-α levels in older *GHR*-KO pigs independent of hypoglycemia.

Bioinformatic analysis of the differentially abundant proteins between *GHR*-KO and WT pigs revealed the pathway “Alpha-amino acid biosynthetic process” as most enriched in CD4^+^ PBMCs of *GHR*-KO pigs. The abundance of glutaminase (GLS), which catalyzes the reaction of glutamine to glutamate [83,84], was increased in CD4^+^ PBMCs of *GHR*-KO pigs. Within CD4^+^ cells of mice, Th17 cells displayed greater expression of GLS compared to Th1 cells, both at the RNA and protein level [83]. It would thus be interesting to analyze if there is a shift towards Th17 cells in CD4^+^ T cells of *GHR*-KO pigs. Proteins with a lower abundance in CD4^+^ PBMCs of *GHR*-KO pigs were cytoplasmatic aspartate aminotransferase (GOT1), phosphoglycerate dehydrogenase (PHGDH), and asparagine synthetase (ASNS).

GOT1 catalyzes the reaction that converts aspartate and α-ketoglutarate to oxaloacetate and glutamate [85,86]. In mice, GOT1 expression was higher in proliferating CD8^+^ effector T cells than in quiescent CD8^+^ memory T cells [87]. The lower abundance of GOT1 in CD4^+^ cells of *GHR*-KO pigs may therefore point to a reduced activation state and a shift towards metabolic quiescence. While GOT1 displayed a lower abundance in CD4^+^ PBMCs of 3-month-old *GHR*-KO pigs, a higher abundance of GOT1 was detected in liver samples of 6-month-old *GHR*-KO pigs [88], suggesting tissue-specific and/or age-related regulatory effects of GH on the abundance of GOT1.

PHGDH is the rate-limiting enzyme in the biosynthesis of serine from intermediates of glycolysis [89]. In CD4^+^ T cells of C57BL/6 mice stimulated with anti-CD3 and anti-CD28 antibodies, *Phgdh* gene expression was found to be higher than in unstimulated controls [90]. The same was true for human CD8^+^ T cells stimulated with anti-CD3 and anti-CD28 antibodies [90]. Therefore, the lower abundance of PHGDH in CD4^+^ cells of *GHR*-KO pigs might indicate a lower activation state.

ASNS converts aspartate and glutamine to asparagine and glutamate in an ATP-dependent reaction [91]. Increased expression of ASNS was found in association with activation of human naïve CD4^+^ T cells and murine CD8^+^ T cells, while lower ASNS expression was found in human CD4^+^ memory Tregs and non-activated naïve mouse CD8^+^ T cells [92,93]. Therefore, the lower abundance of ASNS in CD4^+^ PBMCs from *GHR*-KO pigs suggests a lower activation status and a shift toward a memory or naïve phenotype.

Overall, the changes in protein abundances in CD4^+^ PBMCs from *GHR*-KO pigs indicate an altered amino acid metabolism, which may lead to functional differences in immune cells. Therefore, CD4^+^ T cell subsets should be further investigated, especially CD4^+^ Th17 and Th1 subsets and plasmacytoid dendritic cells. Hypoglycemia in *GHR*-KO pigs might force immune cells to turn to substrates other than glucose (such as amino acids) to meet their energy needs. Enrichment of metabolic pathways associated with amino acid degradation was detected in liver samples from *GHR*-KO pigs [88]. In addition, *GHR*-KO pigs have higher serum levels of urea, the end product of amino acid catabolism [29,94].

Another pathway enriched among the differently abundant proteins in CD4^+^ PBMCs from *GHR*-KO vs. WT pigs was the “Insulin secretion signaling pathway”. The “Insulin secretion signaling pathway” contains proteins that are known to be involved in insulin secretion, but nevertheless, these proteins also have a function in cells that do not secrete insulin, such as immune cells [95]. CD4^+^ PBMCs do not secrete insulin, however, the protein pyruvate carboxylase (PC) included in this pathway, which is involved in insulin secretion, also plays a role in immunometabolism [95,96]. PC was demonstrated to be an important enzyme in the generation of intermediates for the tricarboxylic acid (TCA) cycle in CD8^+^ cytotoxic T cells [95]. Moreover, CD8^+^ effector T cells from mice responding to *Listeria monocytogenes* exhibited a higher protein abundance of PC than naïve CD8^+^ T cells [95,97]. The role of eukaryotic translation initiation factor 4 gamma 1 (EIF4G1), which also clustered to the “Insulin secretion signaling pathway”, in porcine immune cells is unknown, but in humans and mice, EIF4G1 is upregulated in activated T cells compared to Treg and naïve T cells in vitro [98,99]. The proteins PC and EIF4G1 were significantly less abundant in CD4^+^ PBMCs of *GHR*-KO vs. WT pigs, which may suggest a naïve, nonactivated phenotype.

Pathway enrichment analysis of differently abundant proteins in CD4^−^ PBMCs from *GHR*-KO vs. WT pigs revealed “Fatty acid beta-oxidation by acyl-CoA dehydrogenase”. All proteins belonging to this pathway were more abundant in *GHR*-KO than in WT cells, suggesting increased fatty acid beta-oxidation, which has been revealed in several tissues of *Ghr*-KO mice [100]. Acyl-CoA dehydrogenase very long chain (ACADVL) and acyl-CoA dehydrogenase medium chain (ACADM) catalyze the rate-limiting steps of mitochondrial beta-oxidation of very long-chain and medium-chain fatty acids, respectively [101,102,103,104]. CD8^+^ T cells from mice engineered to express higher levels of ACADVL showed higher basal mitochondrial respiration and additional respiratory capacity, indicating improved mitochondrial fitness and response to stress [105]. In contrast, we observed similar basal respiration and spare respiratory capacity in PBMCs from *GHR*-KO and WT pigs. Future studies should examine basal respiration and spare respiratory capacity of cytolytic CD8^+^ T cells separately from CD4^+^ T cells because the higher percentage of naïve CD4^+^CD8α^−^ cells in *GHR*-KO pigs may have decreased the overall basal mitochondrial respiration due to their putative quiescent phenotype.

Another pathway enriched among the differently abundant proteins in CD4^−^ PBMCs from *GHR*-KO vs. WT pigs, which was predicted to be activated in *GHR*-KO cells by IPA analysis, was “Oxidative phosphorylation”. The extent to which immune cells rely on oxidative phosphorylation (OXPHOS) is characteristic of their activation status [86,106]. While human and murine resting naïve and memory T cells and Treg cells exhibit higher rates of OXPHOS than glycolysis, activation of T cells triggers a shift in their metabolism from OXPHOS to high rates of glycolysis and lactate fermentation [86,106]. Thus, activation of this pathway indicates a shift towards a resting non-activated or regulatory phenotype in CD4^−^CD8^+^ T cells of *GHR*-KO pigs. Taken together, the results of pathway analyses pointed to alterations in the immunometabolism of *GHR*-KO pigs.

To functionally analyze the immunometabolism of PBMCs, we measured their oxygen consumption and extracellular acidification—parameters that indicate a cell’s reliance on OXPHOS and glycolysis [42]. Mito stress test was used to measure mitochondrial respiration based on oxygen consumption by the cells and glycolysis based on extracellular acidification [107,108]. With this assay, we detected no significant differences in basal ATP-linked and maximal respiration in PBMCs from *GHR*-KO and WT pigs. Regarding the lack of statistical difference in the Seahorse mito stress test, the sensitivity of the proteomic approach might exceed the sensitivity of the real-time metabolic assay. Moreover, in order to avoid the artificial effects of cell sorting induced by anti-CD4 antibodies, cells were not sorted prior to the mito stress test. While cell sorting is suitable for immediate analytical procedures such as proteomics, it may not be as suitable for functional analysis such as the mito stress test as CD4^+^ cells can be functionally altered by antibodies against CD4 in a time-dependent manner [109,110,111]. We intended to avoid this, especially since *GHR*-KO pigs showed a higher percentage of CD4^+^CD8α^−^ cells in the PBMCs. As a result, CD4^+^ PBMCs, which did not display enhanced OXPHOS in the bioinformatic analysis, were present in the samples of real-time cell metabolic analysis. Thus, it is possible that the results of the mito stress test show similar OXPHOS levels because of the presence of CD4^+^ cells, in contrast to the bioinformatic analysis, where CD4^−^ PBMCs were analyzed separately. Nevertheless, future investigations in CD4^−^ PBMCs of *GHR*-KO pigs with real-time cell metabolic analysis might confirm the higher OXPHOS, as indicated by bioinformatic analysis.

Moreover, the observed alterations in the pathways “Fatty acid beta-oxidation using acyl-CoA dehydrogenase” (increased abundance of ACADVL and ACADM) and “Alpha-amino acid biosynthetic process” (increased abundance of GLS) suggest that PBMCs of hypoglycemic *GHR*-KO pigs may use fatty acids and amino acids as alternative substrates for the generation of precursors for OXPHOS. Substrates for oxidative phosphorylation in the respiratory chain are generated in the TCA cycle [112]. Besides glucose, amino acids and fatty acids also contribute precursors to the TCA cycle, which might compensate for a lack of glucose in the blood of *GHR*-KO pigs by providing α-ketoglutarate and acetyl coenzyme A [112]. The detection of increased serum concentrations of urea, the end product of amino acid catabolism, in *GHR*-KO pigs and *Ghr* KO mice adds evidence to the hypothesis of increased amino acid metabolism in CD4^+^ PBMCs of *GHR*-KO pigs [29,113]. Therefore, further experiments, such as functional assays investigating the substrate preferences of WT and *GHR*-KO PBMCs would be necessary to confirm or reject a difference in the metabolism of *GHR*-KO CD4^+^ PBMCs and possible compensatory mechanisms towards hypoglycemia.

## 5. Conclusions

The aim of this study was to characterize the role of GHR signaling in the cellular immune system. Therefore, a *GHR*-KO pig model characterized by low IGF1 levels and juvenile hypoglycemia was used. Our combined metabolic, proteomics, and functional analysis revealed that relative lymphocyte populations, proliferative capacity, and mitochondrial respiration of PBMCs were not significantly different between WT and *GHR*-KO pigs. However, the percentage of CD4^+^CD8α^−^ cells and serum levels of IFN-α of *GHR*-KO pigs were significantly higher. The results of bioinformatic analysis of significantly differential proteins point to aberrant amino acid and fatty acid metabolism of immune cells of *GHR*-KO pigs. The mechanisms and consequences of these alterations are currently unknown and deserve further investigation.

## Figures and Tables

**Table 1:** Significantly (*p* ≤ 0.05) differentially abundant proteins included in significantly (*p* ≤ 0.05) enriched pathways in CD4^+^ PBMCs of *GHR*-KO pigs (*n* = 4) compared to WT pigs (*n* = 5).

Enriched Pathway	Pathway Genes Total	Proteins
Alpha-amino acid biosynthetic process	58	ASNS, GLS, PHGDH, GOT1, SEPHS2
Insulin secretion signaling pathway	273	EIF4G1, GNA11, PAIP1, PC

Column 1 (enriched pathways) contains the names of enriched pathways in ShinyGO analysis (http://bioinformatics.sdstate.edu/go/ accessed on 8 February 2023) or in IPA analysis (https://digitalinsights.qiagen.com/ accessed on 8 December 2022). Column 2 (pathway genes total) contains the total number of genes in each pathway. Column 3 (proteins) contains the proteins from our dataset clustering to the enriched pathways.

**Table 2:** Significantly (*p* ≤ 0.05) differentially abundant proteins included in significantly (*p* ≤ 0.05) enriched pathways in CD4^−^ PBMCs of *GHR*-KO pigs (*n* = 4) compared to WT pigs (*n* = 5).

Enriched Pathway	Pathway Genes Total	Proteins
Fatty acid beta-oxidation using acyl-CoA dehydrogenase	10	ACADVL, ACADM, IVD, ETFDH
Oxidative phosphorylation	111	ATP5PB, COX5B, NDUFB4, NDUFS2, UQCRH

Column 1 (enriched pathway) contains the names of enriched pathways in ShinyGO analysis (http://bioinformatics.sdstate.edu/go/ accessed on 8 February 2023) or in IPA analysis (https://digitalinsights.qiagen.com/ accessed on 8 December 2022). Column 2 (pathway genes total) contains the total number of genes in each pathway. Column 3 (proteins) contains the proteins from our dataset clustering to the enriched pathways.

**Table 3:** Proteins from mass spectrometry dataset. Significantly (*p* ≤ 0.01) differential.ly abundant proteins quantified with more than 2 peptides in CD4^+^ PBMCs of *GHR*-KO pigs (*n* = 4) compared to WT pigs (*n* = 5).

Accession	Protein	Gene	Ratio	*p*-Value	Peptides Used for Identification
ENSSSCP00000004051	Polypeptide N- acetylgalactosaminyltransferase	GALNT1	0.1	0.0078	4
ENSSSCP00000012194	Nischarin	NISCH	0.5	0.0044	4
ENSSSCP00000007163	D-3-phosphoglycerate dehydrogenase	PHGDH	0.5	0.0089	7
ENSSSCP00000017737	NudC domain containing 3	NUDCD3	0.6	0.0095	5
ENSSSCP00000017707	Protein associated with LIN7 2	PALS2	0.6	0.0081	3
ENSSSCP00000009186	CYFIP related Rac1 interactor A	CYRIA	0.6	0.0056	4
ENSSSCP00000017156	CCR4-NOT transcription complex subunit 9	CNOT9	0.8	0.0033	4
ENSSSCP00000026071	Exportin-2	CSE1L	0.8	0.0026	14

Column 1 (accession) contains the accession number of each identified protein in the Ensembl Database (https://www.ensembl.org/ accessed on 9 December 2022). Column 2 (protein description) contains protein names as listed either on UniProt (https://www.uniprot.org/) or NCBI’s Basic Local Alignment Search Tool (BLAST) (https://blast.ncbi.nlm.nih.gov/Blast.cgi) (both accessed on 9 December 2022). Column 3 (gene symbol) contains the name of the corresponding gene. Column 4 (ratio) contains the fold change in protein abundance. Column 5 (*p*-value) contains the *p*-value as calculated by Student’s *t*-test. Column 6 (Peptides used for quantification) displays the number of unique peptides used for identification.

**Table 4:** Proteins from mass spectrometry dataset. Significantly (*p* ≤ 0.01) differentially abundant proteins, quantified with more than 2 peptides in CD4^+^ PBMCs of *GHR*-KO pigs (*n* = 4) compared to WT pigs (*n* = 5).

Accession	Protein	Gene	Ratio	*p*-Value	Peptides Used for Identification
ENSSSCP00000001946	Fumarylacetoacetase	FAH	1.4	0.0051	13

Column 1 (accession) contains the accession number of each identified protein in the Ensembl Database (https://www.ensembl.org/ accessed on 9 December 2022). Column 2 (protein description) contains protein names as listed either on UniProt (https://www.uniprot.org/) or NCBI’s Basic Local Alignment Search Tool (BLAST) (https://blast.ncbi.nlm.nih.gov/Blast.cgi) (both accessed on 9 December 2022). Column 3 (gene symbol) contains the name of the corresponding gene. Column 4 (ratio) contains the fold change in protein abundance. Column 5 (*p*-value) contains the *p*-value as calculated by Student’s *t*-test. Column 6 (peptides used for quantification) displays the number of unique peptides used for identification.

**Table 5:** Proteins from mass spectrometry dataset. Significantly (*p* ≤ 0.01) differentially abundant proteins, quantified with more than 2 peptides in CD4^−^ PBMCs from *GHR*-KO pigs (*n* = 4) compared to WT pigs (*n* = 5).

Accession	Protein	Gene	Ratio	*p*-Value	Peptides Used for Identification
ENSSSCP00000004071	Interferon-induced protein 44	IFI44	0.5	0.0099	11
ENSSSCP00000024727	Branched-chain-amino-acid aminotransferase	BCAT1	0.6	0.0066	5
ENSSSCP00000028215	Inosine triphosphate pyrophosphatase isoform X3	ITPA	0.6	0.0012	4
ENSSSCP00000025556	Latexin	LXN	0.6	0.0026	6
ENSSSCP00000000794	WASH complex subunit 1	WASHC1	0.6	0.0057	4
ENSSSCP00000008724	Phosducin-like protein 3	PDCL3	0.7	0.0083	6
ENSSSCP00000005105	EH domain-containing protein 4	EHD4	0.7	0.0058	5
ENSSSCP00000008359	RB binding protein 6	RBBP6	0.7	0.0059	8
ENSSSCP00000020456	Tyrosine-protein kinase	BLK	0.7	0.0096	2
ENSSSCP00000000729	Ubiquitin carboxyl-terminal hydrolase	USP5	0.8	0.0077	34
ENSSSCP00000026564	T-complex protein 1 subunit epsilon	CCT5	0.8	0.0061	24
ENSSSCP00000007572	5′-3′ exoribonuclease	XRN2	0.9	0.0082	18

Column 1 (accession) contains the accession number of each identified protein in the Ensembl Database (https://www.ensembl.org/ accessed on 9 December 2022). Column 2 (protein description) contains protein names as listed either on UniProt (https://www.uniprot.org/) or NCBI’s Basic Local Alignment Search Tool (BLAST) (https://blast.ncbi.nlm.nih.gov/Blast.cgi) (both accessed on 9 December 2022). Column 3 (gene symbol) contains the name of the corresponding gene. Column 4 (ratio) contains the fold change in protein abundance. Column 5 (*p*-value) contains the *p*-value as calculated by Student’s *t*-test. Column 6 (peptides used for quantification) displays the number of unique peptides used for identification.

**Table 6:** Proteins from mass spectrometry dataset. Significantly (*p* ≤ 0.01), differentially abundant proteins quantified with more than 2 peptides in CD4^−^ PBMCs of *GHR*-KO pigs (*n* = 4) compared to WT pigs (*n* = 5).

Accession	Protein	Gene	Ratio	*p*-Value	Peptides Used for Identification
ENSSSCP00000008283	Integrin subunit alpha M	ITGAM	2.4	0.0002	7
ENSSSCP00000020286	Runt-related transcription factor 3 isoform X2	RUNX3	2.0	0.0050	2
ENSSSCP00000000739	Condensin complex subunit 1	NCAPD2	1.8	0.0045	5
ENSSSCP00000024566	TRIO and F-actin binding protein	TRIOBP	1.7	0.0037	2
ENSSSCP00000028116	grpE protein homolog 1, mitochondrial	GRPEL1	1.5	0.0039	6
ENSSSCP00000018679	28S ribosomal protein S23, mitochondrial	MRPS23	1.4	0.0038	6

Column 1 (accession) contains the accession number of each identified protein in the Ensembl Database (https://www.ensembl.org/ accessed on 9 December 2022). Column 2 (protein description) contains protein names as listed either on UniProt (https://www.uniprot.org/) or NCBI’s Basic Local Alignment Search Tool (BLAST) (https://blast.ncbi.nlm.nih.gov/Blast.cgi) (both accessed on 9 December 2022). Column 3 (gene symbol) contains the name of the corresponding gene. Column 4 (ratio) contains the fold change in protein abundance. Column 5 (*p*-value) contains the *p*-value as calculated by Student’s *t*-test. Column 6 (peptides used for quantification) displays the number of unique peptides used for identification.

## Data Availability

The mass spectrometry proteomics data presented in this study have been deposited to the ProteomeXchange Consortium via the PRIDE partner repository (https://www.ebi.ac.uk/pride/ accessed on 13 December 2022). Data are publicly available via identifier PXD038772.

## Supplementary Materials

The following supporting information can be downloaded at: https://www.mdpi.com/article/10.3390/biom13040597/s1, Figure S1: Body weights and blood glucose levels of WT and *GHR*-KO pigs; Figure S2: Hierarchical gating strategy for flow cytometry experiments.
