# Supplementary material for: Effects of GHR Deficiency and Juvenile Hypoglycemia on Immune Cells of a Porcine Model for Laron Syndrome
Source: Biomolecules. 2023 Mar 26;13(4):597. doi: 10.3390/biom13040597 (PMC10135491; doi:10.3390/biom13040597)
Supplement: Supplementary file 1 [file biomolecules-13-00597-s001.zip › biomolecules-2256975-supplementary.pdf]

Supplementary figure S1.

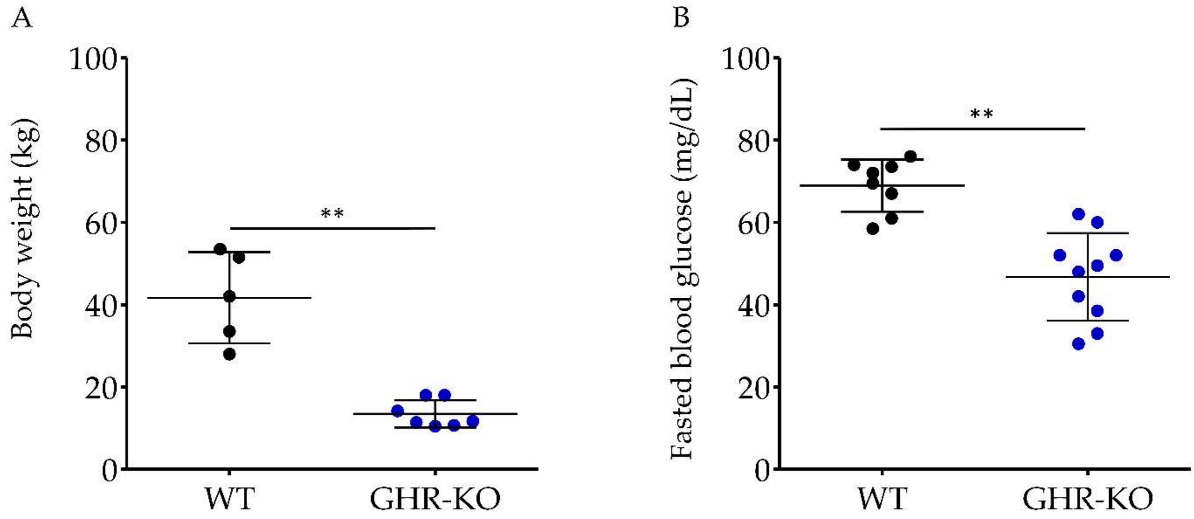

Supplementary figure 1. (**A**) Body weights of wild-type (WT) (black dots, n = 5, 4 females, 1 male) and growth hormone receptor knockout (*GHR*-KO) pigs (blue dots, n = 7, 6 females, 1 male) included in our study significantly differed. A previous study in *GHR*-KO pigs reported a reduction in body weight by approximately 63 % in comparison to WT pigs at an age of three months (Hinrichs et al., 2018). In line with this, *GHR*-KO pigs applied in this study had a body weight of 13.5 ± 1.3 kg in comparison to 41.7 ± 5.0 kg assessed in 5 WT pigs, which equals a reduction in weight by approximately 67%. (**B**) Fasted blood glucose levels of WT (black dots, n = 8, 5 females, 3 males) and *GHR*-KO pigs (blue dots, n = 10, 6 females, 4 males) significantly differed in our study. WT pigs displayed 68.9 ± 6.3 mg/dL blood glucose while *GHR*-KO pigs displayed 46.8 ± 10.6 mg/dL blood glucose, which is in line with the previous characterization of blood glucose levels of these pigs by Hinrichs et al., 2018. Data are shown as mean ± SD; **p < 0.001.

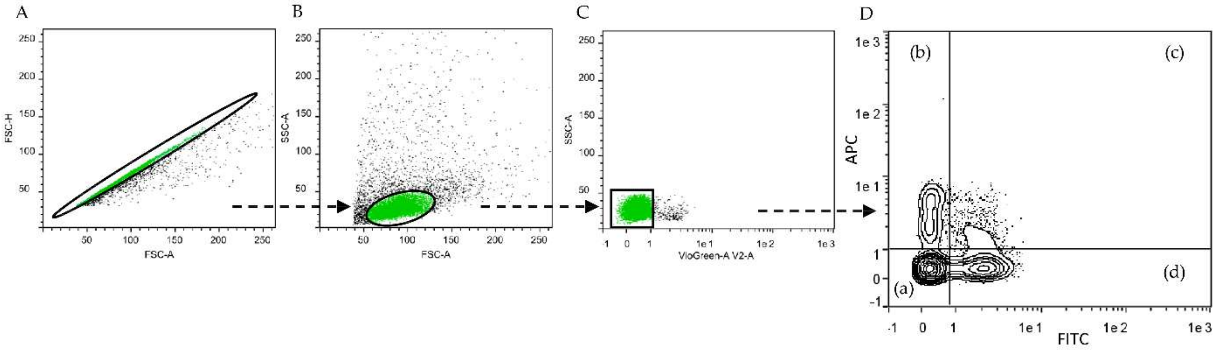

Supplementary figure 2. Hierarchical gating strategy for flow cytometry experiments. PBMC of wt and *GHR*-KO pigs were stained with Viobilty 405/520 fixable dye (Miltenyi Biotech, Bergisch Gladbach, Germany) and measured with MACSQuant Analyzer 10 (Miltenyi Biotech). (**A**) Doublets were excluded by pulse geometry gating comparing area (FSC-A) and height (FSC-H). (**B**) Lymphocytes were identified by gating according to size (FSC-A) and granularity (SSC-A). (**C**) Viable cells were used in further analysis. (**D**) Gating strategy for viable singlets inside the lymphocyte gate. Exemplary gating strategy for (a) negative cells, (b) $CD4^-CD8\alpha^+$ lymphocytes (c) $CD4^+CD8\alpha^+$ activated/memory T cells, (d) $CD4^+CD8\alpha^-$ cells.
